# Dasatinib Inhibits CXCR4 Signaling in Chronic Lymphocytic Leukaemia Cells and Impairs Migration Towards CXCL12

**DOI:** 10.1371/journal.pone.0048929

**Published:** 2012-11-02

**Authors:** Alison M. McCaig, Emilio Cosimo, Michael T. Leach, Alison M. Michie

**Affiliations:** 1 Institute of Cancer Sciences, College of Medical, Veterinary and Life Sciences, University of Glasgow, Glasgow, United Kingdom; 2 Department of Haematology, West of Scotland Cancer Centre, Gartnavel General Hospital, Glasgow, United Kingdom; 3 Department of Haematology, Royal Alexandra Hospital, Paisley, United Kingdom; University of Manitoba, Canada

## Abstract

Chemokines and their ligands play a critical role in enabling chronic lymphocytic leukaemia (CLL) cells access to protective microenvironmental niches within tissues, ultimately resulting in chemoresistance and relapse: disruption of these signaling pathways has become a novel therapeutic approach in CLL. The tyrosine kinase inhibitor dasatinib inhibits migration of several cell lines from solid-organ tumours, but effects on CLL cells have not been reported. We studied the effect of clinically achievable concentrations of dasatinib on signaling induced by the chemokine CXCL12 through its' receptor CXCR4, which is highly expressed on CLL cells. Dasatinib pre-treatment inhibited Akt and ERK phosphorylation in CLL cells upon stimulation with CXCL12. Dasatinib also significantly diminished the rapid increase in actin polymerisation observed in CLL cells following CXCL12 stimulation. Moreover, the drug significantly inhibited chemotaxis in a transwell assay, and reduced the percentage of cells able to migrate beneath a CXCL12-expressing murine stromal cell line. Dasatinib also abrogated the anti-apoptotic effect of prolonged CXCL12 stimulation on cultured CLL cells. These data suggest that dasatinib, akin to other small molecule kinase inhibitors targeting the B-cell receptor signaling pathway, may redistribute CLL cells from protective tissue niches to the peripheral blood, and support the investigation of dasatinib in combination strategies.

## Introduction

It has long been appreciated that chronic lymphocytic leukaemia (CLL) cells are dependent on a number of microenvironmental stimuli for survival and proliferation [1]. The chemokine CXCL12, the ligand for the receptor CXCR4, has a key physiological role in controlling mature B lymphocyte trafficking through germinal centres [2]. CLL cells express high levels of functional CXCR4 [3]; signaling through this receptor reduces spontaneous and drug-induced apoptosis [4] and also facilitates CLL cell migration beneath stromal cells [3]. In addition to promoting chemoresistance, the ability of CLL cells to access and be retained within the bone marrow (BM) and lymph node (LN) microenvironment increases their chance of encountering proliferative signals such as antigenic stimulation of the B cell antigen receptor (BCR), or the T cell factors CD154 (CD40 ligand) and interleukin 4 (IL-4) [5], ultimately resulting in disease progression.

Dasatinib is a tyrosine kinase inhibitor first developed as a ‘second-generation’ ATP-competitive inhibitor of the oncogenic BCR-Abl kinase that characterises chronic myeloid leukaemia, having a potency over three hundred-fold greater than imatinib for the kinase [6]. Dasatinib also inhibits all Src-family tyrosine kinases with an IC_50_ less than 1 nM, and other targets include c-kit (IC_50_ 5 nM), platelet-derived growth factor β (IC_50_ 28 nM), Bruton's tyrosine kinase (BTK; IC_50_ 5 nM) and Tec kinases (IC_50_ 297 nM) [6], [7]. Dasatinib results in significant clinical responses in patients with imatinib-resistant chronic myeloid leukaemia [8], and due to its' multi-kinase targets, research interest has turned to studying the drug in other haematological and solid-organ cancers. Both our group and others have demonstrated that dasatinib inhibits BCR signal transduction and blocks BCR-mediated survival of CLL cells [9], [10], [11]. In solid tumour cell lines and models, including melanoma [12], sarcoma [13], and colon carcinoma [14] dasatinib has been shown to exert significant anti-migratory effects, both *in vitro* and *in vivo*. Therefore, we were interested to assess whether dasatinib disrupted CLL cell migration in response to chemokine stimulation. Here, we show that dasatinib significantly impairs migration of CLL cells toward CXCL12, by inhibiting CXCR4 signaling.

## Methods

### Ethics Statement

Ethical approval was obtained from the West of Scotland Research Ethics Committee. All patients who donated blood samples gave written informed consent in accordance with the Declaration of Helsinki, and samples were anonymised during the study.

### Clinical Samples and Reagents

CLL cells were isolated from peripheral blood as previously described [9]. Clinical details of patients used in these studies are presented in Table 1; none of the patients had received chemotherapy within the preceding 3 months. Immunohistochemistry for ZAP-70 expression and FISH for 11q and 17p deletions was performed by our local clinical pathology laboratory, and results shown in Table 1. Dasatinib was purchased from LC Laboratories (Woburn, MA, USA). As the peak plasma concentration of dasatinib in patients following standard dosing is in the region of 130 nM [15], dasatinib was used at a maximum concentration of 100 nM in experiments.

**Table 1:** Clinical sample details.

Sample I.D.	Age	Sex	Binet Stage	Treated	ZAP-70Status	FISH
3	55	F	A	No	Neg	–
7	73	M	C	Yes	Pos	11q–
12	59	F	A	No	Neg	–
18	63	F	B	Yes	Pos	11q–
21	67	F	C	Yes	Pos	–
32	67	F	B	No	Pos	–
34	64	M	B	Yes	Pos	11q–
35	68	M	B	No	Pos	–
36	76	M	A	Yes	Pos	11q–
41	59	M	A	No	Neg	–
44	63	F	A	No	Neg	–
45	78	M	B	Yes	Pos	–
46	52	F	A	No	Pos	–
50	61	M	C	No	Pos	–
52	78	F	B	Yes	Neg	11q–
64	64	M	C	Yes	Neg	–
62	84	F	A	Yes	ND	–
68	57	F	A	Yes	Pos	–
69	43	M	A	No	Pos	–
70	74	F	C	No	Neg	–

ND = not determined

### Assessment of Actin Polymerisation

CLL cells (×10^6^) were incubated in RPMI-1640 supplemented with 0.5% BSA for 30 min with or without 100 nM dasatinib then stimulated by the addition of 100 ng/ml CXCL12 (PeproTech EC Ltd, London, UK). 100 µl aliquots were removed pre-stimulation, and at 15, 60, 300, and 600 s after stimulation, and fixed/permeabilised in 250 µl Fix/Perm solution (BD Biosciences, Oxford, UK) according to manufacturer's instructions. Cells were then washed in Perm/Wash^™^ buffer (BD Biosciences) and stained with AlexaFluor® 488-labelled phalloidin (Invitrogen Ltd., Paisley, UK) for 10 min. Analysis was performed by acquiring 10,000 events on a FACSCantoII flow cytometer (BD Biosciences).

### Assessment of Chemotaxis

CLL cells (5×10^5^) were incubated in 100 µl RPMI-1640/0.5% BSA media with or without 1, 10, or 100 nM dasatinib for 30 min prior to the assay. Cells were then transferred to the upper chamber of a 6.5-mm diameter Transwell culture insert (Costar®, Fisher Scientific UK.) and placed into wells containing 600 µl media supplemented with or without 150 ng/ml CXCL12, and incubated for 4 hr at 37°C. Thereafter, three 150 µl aliquots were removed from each lower chamber for counting by flow cytometry. For each aliquot the total number of events acquired during 20 s on high flow setting was recorded.

### Pseudoemperipolesis Assay

CLL cells (2×10^6^/ml) were treated with or without with 100 nM dasatinib for 30 min then transferred to collagen-coated wells containing a confluent layer of M2-10B4 fibroblasts. Each experimental condition was set-up in triplicate, and cells were then incubated for 5 hr at 37°C in 5% CO_2_. Following removal of non-migrated cells by thorough washing, the stromal cell layer was trypsinised and stained with an anti-CD19 APC antibody (BD Biosciences) to facilitate isolation of CLL cells by flow cytometry. Pseudoemperipolesed CLL cells were counted by acquiring CD19^+^ events on a FACSCantoII flow cytometer on high flow setting for 30 s. An aliquot of the starting cell population was similarly counted, to enable calculation of the percentage of CLL cells able to transmigrate.

### Assessment of Viability

CLL cell viability was assessed by staining with Annexin V fluorescein isothicyanate and Viaprobe (BD Biosciences) as previously described [9].

### Immunoblotting

CLL cells (3×10^6^/ml) were incubated with or without 100 nM dasatinib in RPMI 1640 containing 0.5% BSA for 30 min, then left unstimulated or further treated with 100 ng/ml CXCL12 at 37°C for 3 or 10 min. Protein lysates were subsequently prepared and analysed by immunoblotting as previously described [9]. All antibodies used in western blotting were sourced from Cell Signaling Technology^®^ (Danvers, MA, USA) apart from phospho-Lyn^396^, which was purchased from Epitomics (Cambridge, MA, USA).

### CLL Cell Proliferation Assays

To analyse cell division, CLL cells were stained with 1 µM CFSE prior to culture. Cells (5×10^5^) were then co-cultured for up to 12 days with NT-L murine fibroblasts stably transfected to express CD154 [16] in media supplemented with 10 ng/ml IL-4 (the CD154/IL-4 system) in the presence or absence of 100 nM dasatinib. Control cells, to which 50 ng/ml colcemid (Sigma Aldrich, Dorset, UK) was added, were included to define the undivided cell population; media and dasatinib was replenished every 72 hr. To assess both cell divisions and absolute cell numbers, cells were stained with an anti-CD19 APC antibody then resuspended in 450 µl buffer to which 50 µl CountBright^™^ beads (Invitrogen Ltd.) was added prior to flow cytometry. To assess cell division, 10,000 CD19^+^ events were recorded for each sample on a FACSCantoII flow cytometer and analysed with FACSDiva software (BD Biosciences). Using the mean fluorescence intensity (MFI) of CFSE in the colcemid control to define the gate encompassing undivided cells, further gates were set for a successive halving of MFI, to include cells that had undergone cell division(s). To determine absolute cell counts, 5000 CountBright^™^ bead events were acquired. CLL cell counts were then determined using a standard formula based on the ratio of CD19^+^ events to beads, following the manufacturer's instructions.

### Statistical Analysis

Data were analysed using the unpaired, 2-tailed, Student *t* test (GraphPad Prism software, La Jolla, CA, USA).

## Results and Discussion

**Figure 1:**
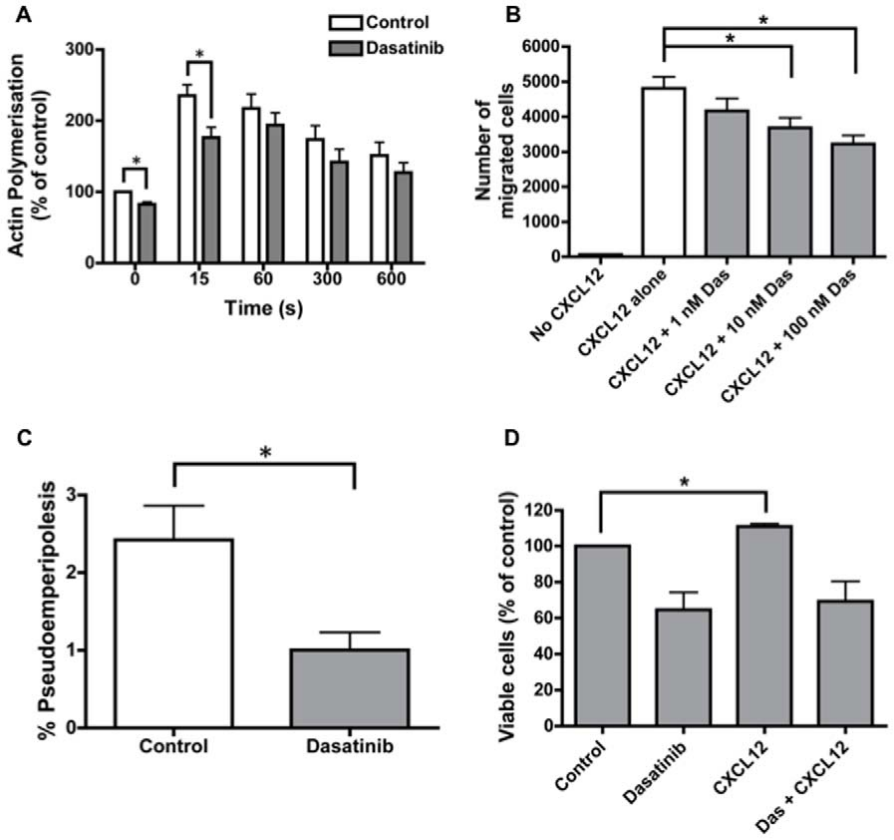
Dasatinib inhibits actin polymerisation and migration of CLL cells in response to CXCL12. **A:** Analysis of the effect of dasatinib on actin polymerization following CXCL12 treatment of CLL cells. Results are expressed relative to the untreated control prior to CXCL12 stimulation. The graph shows the mean (±SEM) values for five independent experiments using different CLL samples (samples 7, 41, 45, 46 and 62). * indicates p<0.05. **B:** The graph shows the effect of increasing concentrations of dasatinib on chemotaxis of CLL cells towards CXCL12. Data are expressed as the average number (±SEM) of migrated cells from five independent experiments (samples 21, 35, 45, 46 and 62). **C:** The graph shows the percentage of CLL cells able to migrate beneath a stromal cell layer (pseudoemperipolesis) in the presence or absence of dasatinib. Results are expressed as the mean (±SEM) percentage of migrated cells in each condition (n = 8; samples 12, 18, 32, 46, 52, 64, 68 and 69). **D:** CLL cells (1×10^6^) were incubated in conditions as shown for 48 hr, then apoptosis was assessed by Annexin/Viaprobe staining. The graph shows the mean (±SEM) viabilities of cells, relative to the viability of untreated control cells (n = 6; samples 12, 36, 41, 44, 45 and 62).

Firstly, we assessed the effect of dasatinib pre-treatment on actin polymerisation in response to CXCL12 stimulation. Dasatinib pre-treatment notably reduced the basal level of actin polymerisation within CLL cells, and also significantly blunted the initial increase in polymerisation following CXCL12 stimulation (Figure 1A). CLL cell migration towards CXCL12 was then assessed in a transwell assay. Dasatinib reduced chemotaxis in a concentration-dependent manner, with 100 nM dasatinib resulting in a mean (±SEM) number of migrated cells of 3229 (±242) as compared to 4812 (±328) in untreated controls (Figure 1B; p<0.001). Moreover, dasatinib significantly reduced the percentage of CLL cells able to migrate beneath the CXCL12-expressing stromal cell line M2-10B4 (Figure 1C; p = 0.02). No significant differences in actin polymerisation or migration were observed between ZAP-70 positive and negative CLL cells in our study. This confirms a previous report that found ZAP-70 positive CLL cells to be more responsive to the chemokines CCL19 and CCL21, but not CXCL12 [17]. As CXCL12 stimulation increases the viability of CLL cells cultured *in vitro* [18], we were also interested to assess whether dasatinib may inhibit the anti-apoptotic effect of CXCL12. CLL cells were cultured for 48 hr in the presence and absence of dasatinib, CXCL12, or both. CXCL12 significantly increased the viability of cultured CLL cells, confirming previous reports (Figure 1D). Dasatinib completely abrogated the anti-apoptotic effect of CXCL12, with cell viability similar to that of cells treated with dasatinib alone (Figure 1D).

**Figure 2:**
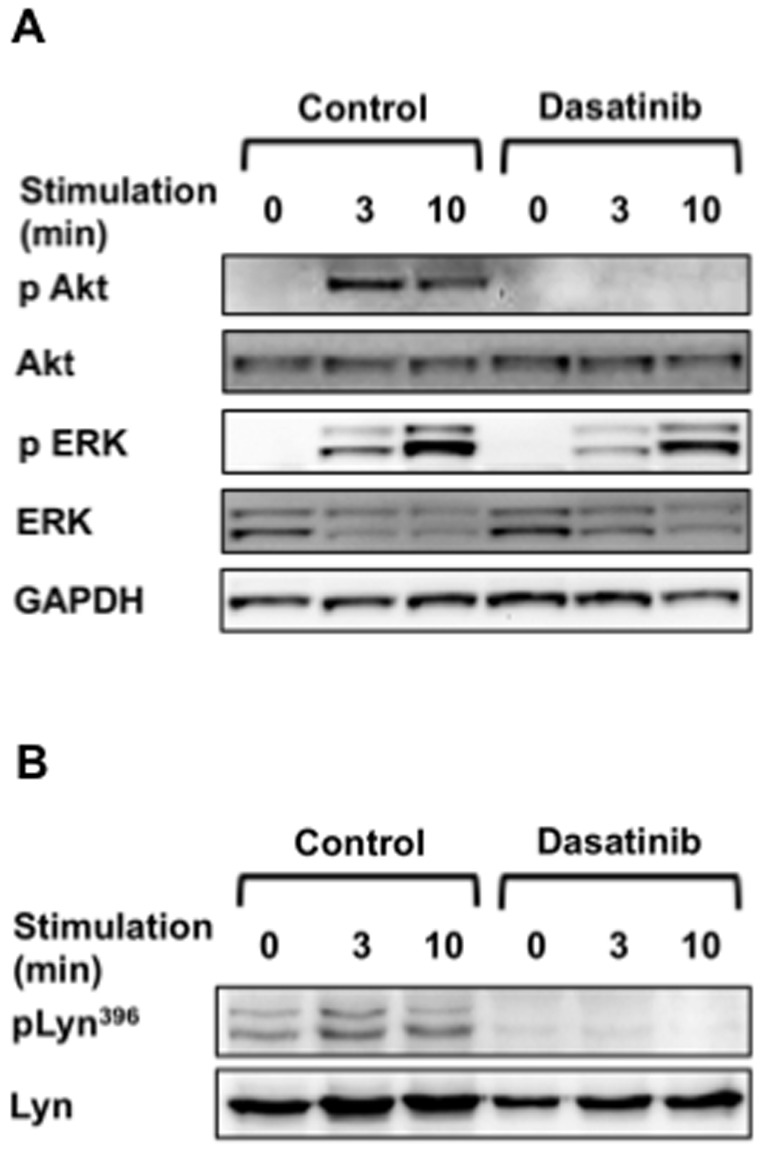
Dasatinib inhibits key signaling events on CXCR4 stimulation. **A:** CLL cells were treated with or without 100 nM dasatinib for 30 min, prior to stimulation with 100 ng/ml CXCL12. Protein lysates were prepared at 3 and 10 min, and immunoblotting performed to determine the level and phosphorylation of Akt and ERK. One representative blot from three independent experiments is shown (samples 35, 45 and 50). **B:** CLL cells were treated as above, and immunoblotting for Lyn^396^ phosphorylation performed. The immunoblot shown is representative of three independent experiments (samples 44, 45 and 70).

As CXCR4 stimulation results in rapid activation of PI-3K and ERK-MAPK in CLL cells [4], [19], [20], we next assessed the activation status of these two signaling pathways following CXCL12 stimulation in the presence or absence of dasatinib. Dasatinib completely abrogated Akt phosphorylation, and partially inhibited ERK activation (Figure 2A). There is substantial evidence indicating that PI-3K/Akt signaling is a key regulator of migration toward CXCL12 in CLL cells. Burger *et al.* showed that the PI-3K inhibitor wortmannin reduced CLL cell migration towards CXCL12, whilst MEK inhibition had no significant effect [3]. More recently, specific PI-3K inhibitors have been shown to inhibit actin polymerisation, chemotaxis, and pseudoemperipolesis in response to CXCL12 [19], [21]. We were next interested to investigate the mechanism by which dasatinib may inhibit Akt phosphorylation in CLL cells in response to CXCL12.

Dasatinib exerts its' pro-apoptotic effects through inhibition of kinases involved in BCR signaling, including Lyn and Syk [7], [9], [10], [11]. Here, we show that dasatinib inhibits Lyn autophosphorylation in the presence and absence of CXCL12 stimulation (Figure 2B). Interestingly, the Src-family kinase Lyn has been demonstrated to control migration of hematopoietic cells, with chemotaxis of BM mononuclear cells from Lyn−/− mice toward CXCL12 impaired by over 75% [22]. Moreover, siRNA knockdown of Lyn in primary CD34^+^ hematopoietic progenitor cells reduced migration toward CXCL12 three to seven-fold over controls [23]. Therefore Lyn inhibition may contribute to the anti-migratory effect of dasatinib in CLL cells. Of note, the Src kinases Lyn and Fyn interact directly with the p85 subunit of PI-3K through their Src homology 3 (SH3) domains in a B cell lymphoma cell line [24], and Lyn co-localizes with PI-3K in HL-60 cells following CXCL12 stimulation [22]. Buchner et al. recently demonstrated CXCL12 stimulation to induce phosphorylation of Syk and Akt in CLL cells, which was abrogated by the small molecule Syk inhibitor R406 [25]. R406 also significantly reduced CLL cell migration toward CXCL12 [25], [26]. Although R406 has a number of off-target effects [27], a recent study demonstrated two novel specific Syk inhibitors, PRT318 and P505–15, to significantly reduce chemotaxis toward CXCL12 or CXCL13 and inhibit pseudoemperipolesis in stromal co-culture experiments [28].

Recently published data suggests that additional dasatinib target kinases may also contribute to the overall anti-migratory effect. BTK activation has been demonstrated following CXCL12 stimulation in a B-cell lymphoma cell line, and the small-molecule BTK inhibitor PCI-32765 blocked CXCL12-induced ERK and Akt phosphorylation in the same cell line [29] and primary CLL cells [30]. Furthermore, PCI-32765 significantly reduced actin polymerization and migration of primary CLL cells toward CXCL12 and CXCL13 [29], [30]. It is notable that the IC_50_ of dasatinib for BTK is 5 nM [7]. In solid tumour cell lines, dasatinib inhibits migration by blocking phosphorylation of Src and the downstream target focal adhesion kinase (FAK) [12], [13], [14]. Of note, Lopez-Guerra *et al.* recently demonstrated phosphorylation of FAK in response to CXCL12 stimulation in CLL cells, and inhibition of Src and FAK by the multikinase inhibitor sorafenib correlated with reduced chemotaxis [31]. In summary, dasatinib targets several key tyrosine kinases that regulate the migration of CLL cells in response to chemokine stimulation, resulting in a significant impairment of chemotaxis.

**Figure 3:**
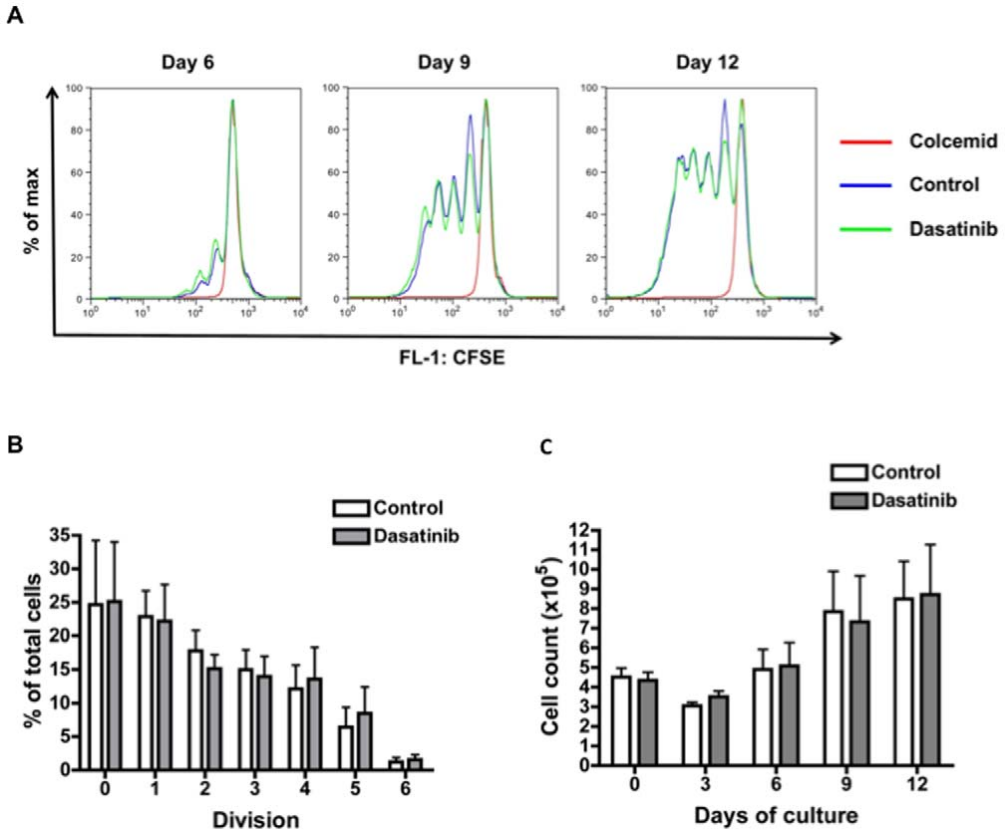
Dasatinib does not inhibit proliferation of CLL cells induced by CD154 and IL-4. **A:** The panels show the assessment of cell division by successive dilution of CFSE at days 6, 9, and 12 of co-culture in the CD154/IL-4 system in one representative experiment. **B:** The graph shows the mean (±SEM) percentage of cells in each cell division at day 12 from six independent experiments (samples 34, 41, 46, 68, 69 and 70). **C:** The graph shows the mean (±SEM) cell counts determined for each condition and time point in all six experiments.

Clinical trials of kinase inhibitors targeting BCR signaling in CLL have confirmed that the anti-migratory effects observed *in vitro* also occur *in vivo* and contribute substantially to overall clinical response. Hoellenriegel *et al.* reported that the PI-3K delta inhibitor CAL-101 induced an early reduction in patient LN size accompanied by a significant lymphocytosis that later resolved on therapy [21], suggesting that CLL cells were mobilised from the protective environment of the BM and LN to the peripheral blood where they were then sensitive to the pro-apoptotic effects of the inhibitor. A transient lymphocytosis is also observed in patients responding to the orally-available Syk inhibitor fostamatinib disodium [32], and the BTK inhibitor PCI-32765 [30] in reported phase I/II trials. To date, there is only one published phase II trial of dasatinib in CLL. In this small study, Amrein *et al*. reported significant nodal responses to be achieved more frequently (9/15 patients) than a reduction in peripheral blood leucocytosis (4/15 patients) [33]. The authors postulated that dasatinib may preferentially induce apoptosis of proliferating CLL cells. Of note, we found no inhibitory effect of dasatinib on proliferation or survival of CLL cells cultured for up to 12 days in the CD154/IL-4 system (Figure 3), an *in vitro* co-culture system that approximates the *in vivo* microenvironment of proliferation centres [34]. These data are consistent with previous reports that the pro-apoptotic effect of dasatinib alone is lost on co-culture with stromal cells expressing CD154 [9], [35], but more importantly, this is the first published report of the effect of dasatinib on CLL cell proliferation. Our data therefore suggest that an *in vivo* anti-migratory effect contributes to the overall clinical response to dasatinib, as observed with the other targeted kinase inhibitors described above. As dasatinib exhibits synergy with both established and novel chemotherapeutic agents in the absence of a protective microenvironment [9] our present data further support the investigation of dasatinib in future combination clinical trials.

## References

[1] Burger JA, Ghia P, Rosenwald A, Caligaris-Cappio F (2009) The microenvironment in mature B-cell malignancies: a target for new treatment strategies. Blood 114: 3367–3375. 19636060 10.1182/blood-2009-06-225326 PMC4969052

[2] Allen CD, Ansel KM, Low C, Lesley R, Tamamura H, et al (2004) Germinal center dark and light zone organization is mediated by CXCR4 and CXCR5. Nat Immunol 5: 943–952. 15300245 10.1038/ni1100

[3] Burger JA, Burger M, Kipps TJ (1999) Chronic lymphocytic leukaemia B cells express functional CXCR4 chemokine receptors that mediate spontaneous migration beneath bone marrow stromal cells. Blood 94: 3658–3667. 10572077

[4] Burger JA, Tsukada N, Burger M, Zvaifler NJ, Dell'Aquila M, et al (2000) Blood-derived nurse-like cells protect chronic lymphocytic leukaemia B cells from spontaneous apoptosis through stromal cell-derived factor-1. Blood 96: 2655–2663. 11023495

[5] Ghia P, Chiorazzi N, Stamatopoulos K (2008) Microenvironmental influences in chronic lymphocytic leukaemia: the role of antigen stimulation. J Intern Med 264: 549–562. 19017179 10.1111/j.1365-2796.2008.02030.x

[6] Lombardo LJ, Lee FY, Chen P, Norris D, Barrish JC, et al (2004) Discovery of N-(2-chloro-6-methyl- phenyl)-2-(6-(4-(2-hydroxyethyl)- piperazin-1-yl)-2-methylpyrimidin-4- ylamino)thiazole-5-carboxamide (BMS-354825), a dual Src/Abl kinase inhibitor with potent antitumor activity in preclinical assays. J Med Chem 47: 6658–6661. 15615512 10.1021/jm049486a

[7] Hantschel O, Rix U, Schmidt U, Burckstummer T, Kneidinger M, et al (2007) The Btk tyrosine kinase is a major target of the Bcr-Abl inhibitor dasatinib. Proc Natl Acad Sci U S A 104: 13283–13288. 17684099 10.1073/pnas.0702654104 PMC1940229

[8] Hochhaus A, Kantarjian HM, Baccarani M, Lipton JH, Apperley JF, et al (2007) Dasatinib induces notable hematologic and cytogenetic responses in chronic-phase chronic myeloid leukaemia after failure of imatinib therapy. Blood 109: 2303–2309. 17138817 10.1182/blood-2006-09-047266

[9] McCaig AM, Cosimo E, Leach MT, Michie AM (2011) Dasatinib inhibits B cell receptor signalling in chronic lymphocytic leukaemia but novel combination approaches are required to overcome additional pro-survival microenvironmental signals. Br J Haematol 153: 199–211. 21352196 10.1111/j.1365-2141.2010.08507.x

[10] Song Z, Lu P, Furman RR, Leonard JP, Martin P, et al (2010) Activities of SYK and PLCgamma2 predict apoptotic response of CLL cells to SRC tyrosine kinase inhibitor dasatinib. Clin Cancer Res 16: 587–599. 20068106 10.1158/1078-0432.CCR-09-1519

[11] Veldurthy A, Patz M, Hagist S, Pallasch CP, Wendtner CM, et al (2008) The kinase inhibitor dasatinib induces apoptosis in chronic lymphocytic leukaemia cells in vitro with preference for a subgroup of patients with unmutated IgVH genes. Blood 112: 1443–1452. 18550857 10.1182/blood-2007-11-123984

[12] Buettner R, Mesa T, Vultur A, Lee F, Jove R (2008) Inhibition of Src family kinases with dasatinib blocks migration and invasion of human melanoma cells. Mol Cancer Res 6: 1766–1774. 19010823 10.1158/1541-7786.MCR-08-0169 PMC2768340

[13] Shor AC, Keschman EA, Lee FY, Muro-Cacho C, Letson GD, et al (2007) Dasatinib inhibits migration and invasion in diverse human sarcoma cell lines and induces apoptosis in bone sarcoma cells dependent on SRC kinase for survival. Cancer Res 67: 2800–2808. 17363602 10.1158/0008-5472.CAN-06-3469

[14] Serrels A, Macpherson IR, Evans TR, Lee FY, Clark EA, et al (2006) Identification of potential biomarkers for measuring inhibition of Src kinase activity in colon cancer cells following treatment with dasatinib. Mol Cancer Ther 5: 3014–3022. 17148760 10.1158/1535-7163.MCT-06-0382

[15] Bristol-Myers-Squibb (2006) Dasatinib (BMS-354825) Oncologic Drug Advisory Committee (ODAC) Briefing Document, NDA 21-986. Bristol-Myers-Squibb Company, Wallingford, CT USA. 21–986.

[16] Hayden RE, Pratt G, Davies NJ, Khanim FL, Birtwistle J, et al (2009) Treatment of primary CLL cells with bezafibrate and medroxyprogesterone acetate induces apoptosis and represses the pro-proliferative signal of CD40-ligand, in part through increased 15dDelta12,14,PGJ2. Leukaemia 23: 292–304. 10.1038/leu.2008.283 18923439

[17] Richardson SJ, Matthews C, Catherwood MA, Alexander HD, Carey BS, et al (2006) ZAP-70 expression is associated with enhanced ability to respond to migratory and survival signals in B-cell chronic lymphocytic leukaemia (B-CLL). Blood 107: 3584–3592. 16332969 10.1182/blood-2005-04-1718

[18] Burger M, Hartmann T, Krome M, Rawluk J, Tamamura H, et al (2005) Small peptide inhibitors of the CXCR4 chemokine receptor (CD184) antagonize the activation, migration, and antiapoptotic responses of CXCL12 in chronic lymphocytic leukaemia B cells. Blood 106: 1824–1830. 15905192 10.1182/blood-2004-12-4918

[19] Niedermeier M, Hennessy BT, Knight ZA, Henneberg M, Hu J, et al (2009) Isoform-selective phosphoinositide 3'-kinase inhibitors inhibit CXCR4 signaling and overcome stromal cell-mediated drug resistance in chronic lymphocytic leukaemia: a novel therapeutic approach. Blood 113: 5549–5557. 19318683 10.1182/blood-2008-06-165068 PMC4580965

[20] O'Hayre M, Salanga CL, Kipps TJ, Messmer D, Dorrestein PC, et al (2010) Elucidating the CXCL12/CXCR4 signaling network in chronic lymphocytic leukaemia through phosphoproteomics analysis. PLoS One 5: e11716. 20661426 10.1371/journal.pone.0011716 PMC2908618

[21] Hoellenriegel J, Meadows SA, Sivina M, Wierda WG, Kantarjian H, et al (2011) The phosphoinositide 3'-kinase delta inhibitor, CAL-101, inhibits B-cell receptor signaling and chemokine networks in chronic lymphocytic leukaemia. Blood 118: 3603–3612. 21803855 10.1182/blood-2011-05-352492 PMC4916562

[22] Ptasznik A, Urbanowska E, Chinta S, Costa MA, Katz BA, et al (2002) Crosstalk between BCR/ABL oncoprotein and CXCR4 signaling through a Src family kinase in human leukaemia cells. J Exp Med 196: 667–678. 12208881 10.1084/jem.20020519 PMC2193994

[23] Nakata Y, Tomkowicz B, Gewirtz AM, Ptasznik A (2006) Integrin inhibition through Lyn-dependent cross talk from CXCR4 chemokine receptors in normal human CD34+ marrow cells. Blood 107: 4234–4239. 16467205 10.1182/blood-2005-08-3343 PMC1895784

[24] Pleiman CM, Hertz WM, Cambier JC (1994) Activation of phosphatidylinositol-3' kinase by Src-family kinase SH3 binding to the p85 subunit. Science 263: 1609–1612. 8128248 10.1126/science.8128248

[25] Buchner M, Baer C, Prinz G, Dierks C, Burger M, et al (2010) Spleen tyrosine kinase inhibition prevents chemokine- and integrin-mediated stromal protective effects in chronic lymphocytic leukaemia. Blood 115: 4497–4506. 20335218 10.1182/blood-2009-07-233692

[26] Quiroga MP, Balakrishnan K, Kurtova AV, Sivina M, Keating MJ, et al (2009) B-cell antigen receptor signaling enhances chronic lymphocytic leukaemia cell migration and survival: specific targeting with a novel spleen tyrosine kinase inhibitor, R406. Blood 114: 1029–1037. 19491390 10.1182/blood-2009-03-212837 PMC4916941

[27] Braselmann S, Taylor V, Zhao H, Wang S, Sylvain C, et al (2006) R406, an orally available spleen tyrosine kinase inhibitor blocks fc receptor signaling and reduces immune complex-mediated inflammation. J Pharmacol Exp Ther 319: 998–1008. 16946104 10.1124/jpet.106.109058

[28] Hoellenriegel J, Coffey GP, Sinha U, Pandey A, Sivina M, et al (2012) Selective, novel spleen tyrosine kinase (Syk) inhibitors suppress chronic lymphocytic leukaemia B-cell activation and migration. Leukaemia 26: 1576–1583. 10.1038/leu.2012.24 PMC5459370 22362000

[29] de Rooij MF, Kuil A, Geest CR, Eldering E, Chang BY, et al (2012) The clinically active BTK inhibitor PCI-32765 targets B-cell receptor- and chemokine-controlled adhesion and migration in chronic lymphocytic leukaemia. Blood 119: 2590–2594. 22279054 10.1182/blood-2011-11-390989

[30] Ponader S, Chen SS, Buggy JJ, Balakrishnan K, Gandhi V, et al (2012) Bruton's tyrosine kinase inhibitor PCI-32765 thwarts chronic lymphocytic leukaemia cell survival and tissue homing in vitro and in vivo. Blood 119: 1182–1189. 22180443 10.1182/blood-2011-10-386417 PMC4916557

[31] Lopez-Guerra M, Xargay-Torrent S, Perez-Galan P, Saborit-Villarroya I, Rosich L, et al (2012) Sorafenib targets BCR kinases and blocks migratory and microenvironmental survival signals in CLL cells. Leukaemia 26: 1429–1432. 10.1038/leu.2011.364 22182921

[32] Friedberg JW, Sharman J, Sweetenham J, Johnston PB, Vose JM, et al (2010) Inhibition of Syk with fostamatinib disodium has significant clinical activity in non-Hodgkin lymphoma and chronic lymphocytic leukaemia. Blood 115: 2578–2585. 19965662 10.1182/blood-2009-08-236471 PMC2852362

[33] Amrein PC, Attar EC, Takvorian T, Hochberg EP, Ballen KK, et al (2011) Phase II study of dasatinib in relapsed or refractory chronic lymphocytic leukaemia. Clin Cancer Res 17: 2977–2986. 21402714 10.1158/1078-0432.CCR-10-2879 PMC3108904

[34] Willimott S, Baou M, Naresh K, Wagner SD (2007) CD154 induces a switch in pro-survival Bcl-2 family members in chronic lymphocytic leukaemia. Br J Haematol 138: 721–732. 17760804 10.1111/j.1365-2141.2007.06717.x

[35] Hallaert DY, Jaspers A, van Noesel CJ, van Oers MH, Kater AP, et al (2008) c-Abl kinase inhibitors overcome CD40-mediated drug resistance in CLL: implications for therapeutic targeting of chemoresistant niches. Blood 112: 5141–5149. 18796631 10.1182/blood-2008-03-146704

